# Iron absorption and oxidant stress during erythropoietin therapy in very low birth weight premature infants: a cohort study

**DOI:** 10.1186/1471-2431-5-29

**Published:** 2005-08-05

**Authors:** James K Friel, Khalid Aziz, Wayne L Andrews, Robert E Serfass

**Affiliations:** 1 Department of Human Nutritional Sciences, University of Manitoba, Winnipeg, Manitoba, R3T 2N2, Canada; 2 Department of Biochemistry, Memorial University, St. John's, Newfoundland, A1B 3X9, Canada; 3 Department of Pediatrics, Memorial University, St. John's, Newfoundland, A1B 3X9, Canada; 4 Department of Preventive Medicine and Community Health, The University of Texas Medical Branch at Galveston, Galveston, Texas, 77555-1109, USA

## Abstract

**Background:**

Iron supplementation may be associated with oxidative stress particularly in premature infants. Our purpose was to examine 1) early supplemental iron during treatment with erythropoietin (EPO) and oxidative stress; 2) enhanced iron absorption during EPO in those infants receiving human milk. Therefore, we determined the effect of erythropoietin plus supplemental iron intakes (4 mg/kg/d) on antioxidant status and iron incorporation.

**Methods:**

Ten very-low-birth-weight infants who were enterally fed and receiving either human milk or formula were followed for 4 weeks during erythropoietin therapy; blood and urine were collected at 3 times; baseline, 2 and 4 weeks later. Once oral feeds commenced the study protocol was initiated. After baseline blood collection, a dose of Fe^57 ^was administered. Two weeks later, a dose of Fe^58 ^was administered as ferrous chloride to determine the effect of human-milk or formula on iron incorporation into RBCs.

**Results:**

Infants started the study at 35 ± 13 days. Incorporation of isotope into RBCs did not differ between formula fed for Fe^57 ^(mean incorporation 8 ± 2.9 n = 3) compared to human-milk fed infants (8.7 ± 5 n = 7) nor for Fe^58 ^(6 ± 2.7 n = 3 *vs*. 8.6 ± 5 n = 7). Tissue damage measured by malondialdehyde in plasma and F-2 – isoprostanes in urine, did not differ by feed or over time. Neither ability to resist oxidative stress/nor RBC superoxide dismutase differed according to feed or over time.

**Conclusion:**

Data suggest that during erythropoietin therapy antioxidant defence in VLBW infants are capable of dealing with early supplemental iron during treatment with EPO.

## Background

Infant formulas (F) have been the prevalent feed for very low birthweight (VLBW, < 1500 g birthweight) infants. More recently, neonatal units have successfully implemented human milk (HM) feeding programs with support groups so that human milk is now routinely fed to the majority of these infants. Rates vary across Canada from ~ 50% in St. John's NFLD to > 90 % in Victoria BC (Derek Matthews, Wayne Andrews, personal communication). Human milk alone may not meet all the nutritional needs of the growing VLBW infant during the first 2 months of life. Therefore, human milk is supplemented with appropriate nutrients [1,2]. Few studies [3,4] have examined iron intakes in VLBW infants receiving human milk. Therefore, the iron needs of this group are poorly defined.

A prevalent condition in VLBW infants during the first months of life is anemia. This "physiologic" anemia may be compounded by non-physiologic mechanisms such as red blood cell loss due to bleeding, hemolysis etc., and frequent blood sampling for clinical purposes [5]. Because the premature infant has a diminished erythropoietin (EPO) response to anemia, the administration of exogenous recombinant EPO has been proposed as a promising therapy. Early trials with EPO alone, reported variable results, which may have been due to limited availability of iron for hemoglobin formation [6-8]. While iron is now commonly provided with EPO, the amounts of iron supplements provided with EPO are not consistent and range from 0–12 mg/kg/d [6,9]. There is little experience with iron administered at these levels in the early life of the neonate and *in only one of the trials with EPO (given intravenously as well as orally) has the safety of supplemental intakes of iron been examined *[9]. Further, 1) little data is available on iron absorption in these infants 2) no distinction is made between breast-fed and formula fed infants in terms of possible differences in iron absorption related to the large amounts of iron provided as supplements during EPO. If iron absorption is indeed greater in human milk fed than formula-fed infants [10,11], then human milk fed VLBW infants may be at increased risk for problems associated with iron excess, particularly production of free radicals [12].

We used erythrocyte incorporation of an iron stable isotope as a surrogate for iron absorption in the intestinal tract (6, 9, 10). We assume that in identical conditions prompt erythrocyte incorporation of iron would account for the same percentage of newly absorbed iron by all infants in the study.

Our hypotheses were: 1) intakes of early supplemental iron during treatment with EPO would stress antioxidant defenses and lead to oxidant damage 2) iron absorption during EPO therapy plus iron supplements would be elevated with HM feeding. This study was necessary as the effect on oxidative stress during EPO therapy has not been reported nor has the possible increase in absorption of iron in human milk feeding been examined during EPO. We did not find any effect of HM on iron absorption nor an increase in oxidative stress during EPO.

## Methods

The study included 10 VLBW infants who received EPO plus iron (2–4 mg/kg/d) as part of their normal clinical care. The study protocol was approved by the Memorial University Ethics Committee and informed written permission was obtained from parents/guardians. The baseline period was that time before the administration of EPO and iron isotopes. EPO treatment began when infants were tolerating 75% of feeds (non-iron fortified formula or human milk) by the enteral route. On two separate occasions, before commencement of EPO treatment, and again after 2 weeks of EPO treatment stable non-radioactive isotopes of iron (^57^Fe, ^58^Fe) were administered enterally. At baseline and again at 2 and 4 weeks of treatment, a blood sample was collected by venipuncture and a 12-hour urine sample was collected. We thereby tested the two hypotheses that firstly, early supplemental iron intakes (2–4 mg/kg/d) result in oxidant stress, and that secondly, incorporation of ingested iron is affected by type of feed (human milk or formula) that the infant receives.

### Subjects and feedings

Eligibility required birth weight < 1500 g, < 34 weeks gestation receiving oral or gastric feeds (75%), stable respiratory status as defined by FIO_2 _≤ 30%, and a mean airway pressure of 8 cm of water or less, if on assisted ventilation. Infants were not eligible if they received transfusions within the previous week or during the 4 week study period, had any major congenital defect, liver disease, necrotizing enterocolitis, grade III or IV intracranial hemorrhage or culture-proven sepsis.

All infants received parenteral nutrition with no added iron before study entry. During the study, 3 infants received formula (Similac Special Care, Ross Products Division, Abbott Laboratories, Columbus, OH) and the remaining 7 infants received their own mother's milk fortified with Enfamil Human Milk Fortifier (Mead Johnson Nutritionals, Evansville, IN). No iron or vitamin supplementation were given on the day of isotope administration, otherwise management of nutrition was under the direction of the medical team.

Beginning at study entry, r-HuEPO (R.W. Johnson Pharmaceutical Research Institute, Raritan, NJ) was administered S.C. in a dose of 200 U/kg 3 times a week for six weeks. Iron was given concomitantly in graded doses of 2 mg/kg/d wks 1 and 2 and 4 mg/kg/d, wks 3 and 4.

### Administration of ^57^Fe and ^58^Fe test doses

At baseline (^57^Fe dosing) and again on d 14 (^58^Fe dosing) each infant received one dose with a regular feed (as described previously). A 24 hour equilibration time was used and is sufficient to allow isotope to mix with native iron in human milk [10]. The average daily volume of milk at baseline was 148 ± 20 mL/kg and at week 2, 158 ± 18 mL/kg (x ± SD).

Enriched ^57^Fe (95 weight % ^57^Fe, Atomergix Inc. Farmingdale, NY), and ^58^Fe (93.05 weight % ^58^Fe, Cambridge Isotope Labs, Andover, MA) were converted to ferrous chloride as described previously [13]. Enriched oxides were dissolved in a small amount of Aqua Regia (HCl: HN0_3_, 3:1), diluted, pH adjusted to 5 with NaOH, brought up to a volume of 20 mL with saline and filtered through a 0.2 um filter and stored in glass vials until use. Before use, each vial was checked for purity from pyrogens with a test kit (Limulus Ambeocyte Lysate Test Kit: Whittaker Bioproducts, Walkersville, MD).

To prepare the enriched formulas, an amount of solution which provided approximately 1800 ug ^57^Fe/kg or 450 ug ^58^Fe/kg was added directly to the milk using a pre-weighed syringe. ^57^Fe was equilibrated with vitamin C overnight in milk alone. ^58^Fe was mixed in milk with vitamin C and a solution of natural abundance ferrous sulfate (Fer-In-Sol, Mead Johnson, Inc., Evansville, IN). The final iron content of the first feed with ^57^Fe was 0.2 mg/kg. The final iron content of the latter feed was set to 4 mg/kg made up from ^58^Fe, endogenous iron in the formula as labeled, (human milk was considered to have negligible iron) with the majority of iron from ferrous sulfate. This final dosage matched the usual treatment dose (4 mg/kg/d) provided with EPO at this time period. Milk administered by bottle was flushed with 5 mL of a 10% glucose solution.

### Laboratory methods

Hemoglobin (Hgb) concentration was determined in whole blood by Coulter Counter (Coulter Electronics, Inc., Hialeah, FL). Whole blood was centrifuged and plasma removed. Red blood cells (RBCs) were washed 3 times in isotonic saline and both plasma and RBCs frozen at -80°C until analysis.

For isotopic analyses, iron was obtained from RBCs according to Fomon et al. [14]. Two week study periods were used as 2 weeks is the time required for enrichment of RBCs (14). For each of 10 infants, 3 separate samples were analyzed for a total of 20 estimations of iron absorption, plus 10 baseline measurements. Briefly, after microwave digestion with concentrated nitric acid (HNO_3_), in closed vessels, iron was selectively extracted with xylene that contained thenoyltrifluoroacetone and processed as reported previously [15]. Samples were analysed for ^58^Fe/^54^Fe and ^57^Fe/^54^Fe ratios by inductively coupled plasma mass spectrometry (ICP-MS, Plasma Quad3, Thermo Elemental, Franklin, MA US).

The quantity of administered ^57^Fe and ^58^Fe label incorporated into erythrocytes 14 days after each dose was calculated as described previously [14];

Total circulating iron was estimated as; Fe _circ _= BV * Hgb * 3.47. BV is blood volume, assumed to be 0.085 L/kg [16], Hgb is hemoglobin concentration in g/L, and 3.47 is the mg/g concentration of iron in Hgb. The following assumption was made; of the isotropic iron that is absorbed, by 14 d, 80% is incorporated into erythrocytes [14].

All glass and plastic beakers, flasks and pipettes were acid washed and only distilled deionised water was used throughout as previously reported [17]. Iron concentrations were determined by atomic absorption spectrometry (17). NIST standard reference material spectrophotometric grade iron was run as a reference standard and to determine a normalization factor applied to each sample. To measure oxidant stress in plasma, malondialdehyde (MDA) was measured by HPLC [18]. Total radical antioxidant protection (TRAP) as a measure of the ability to resist oxidative stress was measured using an oxygen electrode (YSI Model 5331) according to Wayner et al. [19]. In erythrocytes, superoxide dismutase (SOD) and catalase (CAT) were determined spectrophotometrically [20,21]. F_2_-Iso-prostanes as a measure of oxidant stress in urine were measured using a commercially available ELISA kits (Oxford Biomedical Research Inc., Oxford, MI). Information on oxygen exposure, medications, transfusions daily nutrient intakes and other data was recorded from nursing records.

Differences in descriptive data for study subjects were assessed by Student's *t *tests (SPSS- version 10, SPSS Inc., Chicago, IL). Pearson Correlation Coefficients were determined between iron absorption and study weight, birthweight, gender and gestational age. Differences in iron incorporation by type of feed and oxidative stress were measured by both parametric and nonparametric tests with consistent results.

Sample size was determined from measurements taken from previous studies in similar infants (13, 17) based on iron incorporation. We expected absorption in the F group to be 10 % with an SD of 5 %. In the HM fed group we expected absorption of 30% with an SD of 5 based on our earlier work (13, 17). Thus with an expected difference of 20 %, a power of 0.8 and an alpha of 0.05 we would need 5 subjects in each group. Significance for all assays was assigned to p < 0.05 (two-tailed).

## Results

Infants weighed 1046 ± 212 g at birth and were 28 ± 2 weeks gestational age (x ± SD: n = 10). All infants gained approximately 30 g/d in weight during the 4 week study period. For these infants: age at study entry was 35 ± 13 days (range 22–68 days); weight at study entry was 1363 ± 217 g; week 2, 1708 ± 312 g; and week 4, 2167 ± 283 g. HGB at baseline (87 ± 12 g/L) week 2 (89 v 11 g/L) and week 4 (99 ± 12 g/L) increased significantly between weeks 2 and 4. There was no difference in birth weight, gestational age or weight at baseline between HM (n = 7) fed infants and F (n = 3) fed infants.

Data for each subject is presented in Table 1. Incorporation into RBCs of ^57^Fe after the first 14 days of the study period was 8.7 ± 5 % for HM fed infants and 8 ± 3 % for F fed infants and did not differ. Incorporation of ^58^Fe from days 14 to 28 of the study period into RBCs was 8.6 ± 5 % for HM fed infants and 6.0 ± 2.7 % for F fed infants and did not differ. Mean percent relative standard deviation for all 30 samples including baseline that were analyzed for isotopic enrichment was 0.62 ± 0.10 for ^57^Fe/^54^Fe ratios and 0.77 ± 0.29% for ^58^Fe/^54^Fe ratios.

**Table 1:** Oxidant status and iron incorporation in VLBW Infants during treatment with EPO plus iron

	Baseline	Week 2	Week 4
PLASMA TRAP (umol/L	492 ± 345^1^	510 ± 316	438 ± 293
PLASMA MDA (umol/L)	0.35 ± 0.47	0.33 ± 0.24	0.42 ± 0.48
URINE F_2 _-Isoprostanes (ng/mL)	19 ± 14	29 ± 30	30 ± 19
RBC SOD (U/mL)	3.1 ± 2	4.0 ± 2.3	3.8 ± 2.1
RBC CAT (U/mL)	62 ± 19	68 ± 25	75 ± 25
INCORP (%)	______	8.5 ± 4.2	7.8 ± 4.4

^1^means ± S.D.

Energy and protein intake and volume of milk fed were at base line; 103 ± 18 kcals/kg, 2.6 ± 0.6 g/kg and 139 ± 17 ml/kg. At week 2, energy intake was 134 = 30 kcal/kg, protein intake was 3.1 ± 0.7 g kg and volume of milk fed was 158 ± 10 ml/kg.

Data for plasma TRAP, MDA, urinary F-2-isoprostanes, RBC SOD and CAT are presented in Table 1. As there was no difference between feeding groups, data were pooled. Control values for healthy adults and premature infants of similar gestational ages not receiving EPO from our laboratory are: for TRAP 964 ± 240, umol/L (adult, n = 23); MDA 0.35 ± 0.17 umol/L (premature infant n = 36) 0.38 ± 0.16 (adult, n = 13); urinary F_2 _isoprostanes 28 ± 13 ng/mL (premature infant, n = 24); RBC SOD, 3.2 ± 1.8 U/ml (premature infant, n = 33) 1.2 ± 0.3 (adult n = 10); RBC CAT 68 ± 14 U/mL (premature infant, n = 30) 12 ± 20 u/mL (adult, n = 20). As well, each infant at baseline served as their own control for later analyses.

Measures of antioxidant status and oxidant stress did not correlate with birth weight, gestational age, Apgar scores, and volume of feed nor days of oxygen exposure.

## Discussion

### Iron isotopes

While our sample size is small (n = 10 infants), we were able to obtain 3 measurements from each infant serving as their own control. As well, the sample size is similar to isotope studies reported in other infants (6, 10). We had hoped to enrol at least 5 infants in each group as our power analysis suggested, but this did not occur, thereby increasing the chance of a Type II error. From this data, we did not find any difference in incorporation of iron isotopes into RBCs between VLBW infants who were receiving either human milk or premature formula (Table 1). In a previous experiment [13] we found that isotope incorporation could be affected by zinc intakes in these infants in a non-meal situation. We chose however to examine iron absorption as it would occur naturally in the NICU, which is during the fed state. However, we are left too speculate on the comparisons between feeding groups because of the small sample size. We believe there is merit in presenting this data as we originally hypothesized large differences which clearly did not occur.

While it is known that food components can enhance iron absorption, in general iron absorption decreases with food intake [22]. In-between feedings iron absorption has been shown to range from 32 to 42% exhibiting wide variation [4,23,24]. With meals, absorption of stable and radioactive iron isotopes has been reported to be from 2.8 to 15.3%, a range that our results fall within [4,25-27]. It has been suggested that iron absorption by premature infants is unregulated by the level of iron intake [4].

Fomon [14] found that the mean RBC Fe^58 ^incorporation was 7.8% for HM fed infants compared to 4.4% for formula fed infants even when the isotope was fed between meals. He suggested that his difference was due to the greater quantities of inhibitors present in the intestine of formula fed infants. Whether or not the assumed greater absorption of Fe in HM is due to enhancement by HM or lack of inhibitors present in formula feeding is still unresolved. This latter report was one of the studies that generated our hypothesis however; we did not find the same results as Fomon [14] as we studied VLBW infants who received 4 times the iron dose as did full-term infants in the latter study. As well, Fomon [14] suggested that greater iron incorporation into RBCs in HM fed infants made up for lower Fe content in HM whereas in our study during EPO treatment, the quantity of exogenous iron added to feedings was so large as to mask any contribution from milk feedings. The finding could also be a Type II error due to the uneven sample size.

However the important finding in the current study is that RBC incorporation of iron isotope does not appear to be different in HM fed infants as compared to formula fed infants in any significant way. Earlier reports suggest an order of magnitude difference between HM and formula fed infants (10, 11, 13) particularly in our earlier study where one HM fed infants had an absorption of 35% of the isotopic dose. Thus even the 1–2% possible difference in our study that may have been found with a larger sample size does not indicate a concern for over absorption of iron in HM fed premature infants when larger does of iron are given with EPO therapy. Therefore concern of potential iron excess or increased free radical generation with EPO plus iron in HM fed infants does not appear warranted.

### Oxidant stress

There has only been one study to date examining the effects of EPO on potential free radical damage [9] in VLBW infants and one report in adults [28]. Pollack [9] reported a transient rise in MDA after intravenous iron infusion. The present study with a more comprehensive assessment did not find similar results either in MDA or F_2 _isoprostane levels. It is well known that iron may cause oxidative damage however much of the data is from *in vitro *assessment. As there is a substantial increase in iron utilization during treatment with EPO, it may be that iron is sequestered and not available for free radical chemistry adults, EPO therapy was studied in hemodialysed patients with and without iron [28]. EPO plus iron did not lead to increased MDA levels however SOD, GHSPx and CAT increased in this group of patients. In the present study, we found a trend (p = 0.07) to increased CAT levels. The authors in the latter study suggested that increased enzyme activity levels might be due to an increased number of reticulocytes or a stimulation of enzyme synthesis in young RBC by reactive oxygen species. Differences in these latter studies may be due to different populations and the much longer time of treatment (>6 months) in the adult population. In the current study, lack of evidence of lipid peroxidation suggests that increased CAT values are physiological and well within the infants' ability to handle the current amounts of iron given with EPO.

Recent work has also suggested that EPO plus early iron does not affect anthropometry, need for rehospitilization, transfusion after discharge nor developmental outcome (29). Thus longer term data supports our short term data that at least from the point of oxidative stress premature infants can cope with early supplemental iron plus EPO.

## Conclusion

a) When EPO therapy is used, particularly with iron intakes up to 4 mg/kg/d, our data suggests that, in regards to oxidative stress, the process appears to be safe.

b) Human milk feeding during early supplemental iron during treatment with EPO does not enhance iron absorption. Increased iron absorption would have been a concern in the VLBW population.

## Abbreviations

CAT Catalase

EPO Erythropoietin

F Formulas

^57^Fe Iron stable isotope mass 57

^58^Fe Iron stable isotope mass 58

HCL Hydrochloric acid

Hgb Hemoglobin

HM Human milk

HNO_3 _Nitric acid

ICP-MS Inductively coupled mass spectrometry

INCORP Iron Incorporation (%)

MDA Malondialdehyde

NIST National Institute of Standards and Technology

RBC Red blood cell

SOD Superoxide dismutase

TRAP Total Radical Trapping Antioxidant Parameter

VLBW Very low birth weight

## Competing interests

The author(s) declare that they have no competing interests.

## Authors' contributions

RES was responsible for isotopic analyses. KA and WLA were in addition responsible for patient recruitment. JKF originated the study, obtained funding and was responsible for day to day maintenance of the study protocol. All authors read and approved the final manuscript.

## Pre-publication history

The pre-publication history for this paper can be accessed here:


